# Supplementary material for: A Likelihood Approach for Real-Time Calibration of Stochastic Compartmental Epidemic Models
Source: PLoS Comput Biol. 2017 Jan 17;13(1):e1005257. doi: 10.1371/journal.pcbi.1005257 (PMC5240920; doi:10.1371/journal.pcbi.1005257)
Supplement: S1 File — (TAR.GZ) [file pcbi.1005257.s014.tar.gz › HSPH_Online-SI-Revision/MSS10/n10-severe/n10-severe_NEW_table4.pdf]

1	R0	{1.68952, 11.8402, 1.}	{1.7434, 11.5667, 2.}	{{1.7434, 11.5667, 2.}, {1.7434, 11.5667, 2.}}	{3.18914, 2.30994, 100.}	inf	584.	886.391	{861.896, 908.252}	51.7794	0.181107	newly	319.	473.	{447., 498.}	48.2759	0.170741	Reff	0.653675	0.621803	{0.61694, 0.625982}	4.87587	ar	6872.	7437.09	{7348., 7513.}	8.22308
2	R0	{1.71739, 9.62661, 0.1}	{1.61188, 8.7843, 3.}	{{1.61188, 8.7843, 3.}, {1.61188, 8.7843, 3.}}	{6.14372, 8.74986, 2900.}	inf	487.	351.406	{326.187, 374.775}	27.8428	0.142254	newly	307.	241.42	{219., 262.}	21.3616	0.10541	Reff	0.669954	0.65065	{0.64683, 0.654684}	2.88138	ar	6874.	6509.83	{6421., 6598.}	5.29779
3	R0	{1.48936, 6.23975, 0.1}	{1.47651, 5.4396, 3.}	{{1.47651, 5.4396, 3.}, {1.47651, 5.4396, 3.}}	{0.86282, 12.8234, 2900.}	inf	196.	158.217	{133.385, 178.757}	19.5376	0.0971073	newly	176.	166.69	{141., 191.}	9.96023	0.0458601	Reff	0.69285	0.692451	{0.68902, 0.695937}	0.314567	ar	5666.	5629.83	{5509., 5736.}	1.36834
4	R0	{1.62394, 7.51135, 1.}	{1.58886, 7.29751, 1.}	{{1.58886, 7.29751, 1.}, {1.58886, 7.29751, 1.}}	{2.15991, 2.84689, 0.}	inf	314.	346.829	{321.145, 373.474}	10.5351	0.0428743	newly	272.	282.48	{258., 307.}	6.47794	0.0270741	Reff	0.684491	0.66449	{0.66009, 0.668463}	2.92194	ar	6417.	6373.84	{6264., 6466.}	1.16721
5	R0	{1.53632, 8.57652, 1.}	{1.47717, 5.11366, 2.34}	{{1.47488, 4.06661, 0.}, {1.47651, 5.4396, 3.}}	{3.84981, 40.3761, 178.}	inf	366.	158.946	{127.823, 189.259}	56.5722	0.36724	newly	274.	177.6	{140., 212.}	35.1825	0.192878	Reff	0.697643	0.701369	{0.69631, 0.705519}	0.616381	ar	6260.	5590.1	{5479., 5705.}	10.7013
6	R0	{1.47166, 5.7434, 0.1}	{1.47539, 5.15861, 2.4}	{{1.47488, 4.06661, 0.}, {1.47651, 5.4396, 3.}}	{0.351466, 10.1819, 2340.}	inf	206.	189.689	{133.496, 231.435}	14.7235	0.0734498	newly	216.	209.86	{164., 255.}	13.213	0.0607115	Reff	0.704484	0.708671	{0.701708, 0.715961}	0.762574	ar	5650.	5596.47	{5403., 5783.}	2.06885
7	R0	{1.48179, 8.87369, 0.1}	{1.38815, 6.71734, 0.}	{{1.38815, 6.71734, 0.}, {1.38815, 6.71734, 0.}}	{6.31916, 24.3005, 100.}	inf	206.	132.179	{113.911, 153.309}	35.8357	0.195952	newly	142.	119.73	{99., 139.}	17.1479	0.0845292	Reff	0.774828	0.736113	{0.733141, 0.73859}	4.9966	ar	5233.	5016.71	{4916., 5111.}	4.13319
8	R0	{1.53669, 6.47807, 0.1}	{1.48918, 5.20634, 3.01}	{{1.47651, 4.79544, 3.}, {1.50982, 5.4396, 3.}}	{3.10186, 19.6313, 2910.}	inf	235.	142.164	{109.014, 169.641}	39.5048	0.223878	newly	203.	154.14	{122., 183.}	24.1872	0.125785	Reff	0.682444	0.675178	{0.665501, 0.687258}	1.46632	ar	5934.	5742.52	{5642., 5826.}	3.22986
9	R0	{1.57227, 6.20989, 0.1}	{1.53211, 5.12025, 3.64}	{{1.50982, 4.79544, 3.}, {1.54465, 5.30296, 4.}}	{2.55414, 17.5468, 3540.}	inf	357.	259.214	{220.062, 298.82}	27.3911	0.142295	newly	361.	287.76	{250., 336.}	20.3712	0.101892	Reff	0.71271	0.709461	{0.699873, 0.717548}	0.822745	ar	6136.	5896.7	{5737., 6044.}	3.90124
10	R0	{1.54936, 6.50938, 0.1}	{1.58989, 5.10138, 4.}	{{1.59034, 5.09935, 4.}, {1.59034, 5.09935, 4.}}	{2.62173, 21.6303, 3900.}	inf	238.	160.302	{129.372, 197.827}	32.6461	0.176837	newly	209.	173.87	{132., 213.}	18.0909	0.0913141	Reff	0.623308	0.651964	{0.645946, 0.656736}	4.59746	ar	6275.	6171.36	{6075., 6250.}	1.81068
11	R0	{1.42179, 3.18601, 0.1}	{1.43965, 2.60601, 1.}	{{1.43965, 2.60601, 1.}, {1.43965, 2.60601, 1.}}	{1.25644, 18.2045, 900.}	inf	53.	35.8411	{12.6491, 59.6297}	40.6859	0.279982	newly	90.	64.25	{25., 112.}	39.8333	0.273462	Reff	0.684876	0.695952	{0.692035, 0.699099}	1.61713	ar	5322.	5240.06	{5162., 5323.}	1.66704
12	R0	{1.48035, 6.46183, 0.1}	{1.47607, 5.28568, 2.67}	{{1.47488, 4.06661, 0.}, {1.47651, 5.4396, 3.}}	{0.289136, 18.2015, 2592.}	inf	313.	237.985	{180.681, 271.67}	23.9663	0.124505	newly	284.	256.44	{216., 292.}	11.4648	0.054783	Reff	0.695468	0.704527	{0.699572, 0.710832}	1.30253	ar	5793.	5698.51	{5567., 5832.}	1.99051
13	R0	{1.75755, 8.53854, 1.}	{1.67476, 7.30504, 1.08}	{{1.67405, 7.28757, 1.}, {1.67405, 7.28757, 1.}}	{4.71036, 14.4463, 8.}	inf	415.	388.134	{355.859, 416.85}	6.93059	0.0317882	newly	276.	309.95	{279., 339.}	12.8804	0.0517146	Reff	0.648009	0.621983	{0.616912, 0.627123}	4.01623	ar	6832.	6817.3	{6726., 6924.}	0.95404
14	R0	{1.47355, 4.89451, 0.1}	{1.48612, 4.53515, 1.65}	{{1.47488, 4.06661, 0.}, {1.50982, 5.4396, 3.}}	{0.887985, 11.1286, 1640.}	inf	140.	114.25	{73.9714, 150.883}	23.0356	0.121303	newly	163.	136.69	{96., 174.}	19.9202	0.103511	Reff	0.670023	0.679556	{0.669758, 0.690058}	1.49439	ar	5710.	5646.8	{5514., 5775.}	1.66865
15	R0	{1.62071, 5.98665, 0.1}	{1.67266, 5.65432, 3.92}	{{1.65865, 5.3704, 2.}, {1.69756, 6.15907, 5.}}	{3.20538, 7.62484, 3820.}	inf	218.	222.767	{157.729, 304.776}	22.5247	0.0962079	newly	197.	213.35	{161., 275.}	18.8477	0.0762399	Reff	0.578593	0.596245	{0.590954, 0.60145}	3.05076	ar	6635.	6729.03	{6577., 6916.}	1.84099
16	R0	{1.67268, 7.7256, 1.}	{1.68297, 7.5059, 2.}	{{1.68297, 7.5059, 2.}, {1.68297, 7.5059, 2.}}	{0.615065, 2.8438, 100.}	inf	248.	147.828	{130.039, 164.434}	40.3921	0.226496	newly	184.	113.54	{95., 130.}	38.2935	0.212486	Reff	0.552988	0.573248	{0.570423, 0.57622}	3.66372	ar	6947.	6780.85	{6719., 6839.}	2.39168
17	R0	{1.67805, 11.1417, 1.}	{1.47369, 5.256, 2.61}	{{1.454, 4.00571, 0.}, {1.47651, 5.4396, 3.}}	{12.1784, 52.8258, 187.}	inf	598.	233.459	{181.529, 274.811}	60.96	0.413369	newly	353.	259.93	{215., 303.}	26.3654	0.136465	Reff	0.775091	0.734406	{0.728827, 0.74041}	5.24906	ar	6625.	5536.49	{5345., 5677.}	16.4303
18	R0	{1.66743, 9.99046, 0.1}	{1.60857, 8.77201, 3.9}	{{1.6082, 8.77064, 3.}, {1.6082, 8.77064, 4.}}	{3.53027, 12.1961, 3800.}	inf	590.	509.771	{489.016, 531.361}	13.5981	0.0637555	newly	394.	355.69	{333., 379.}	9.72335	0.0449828	Reff	0.682146	0.670969	{0.667363, 0.674243}	1.63842	ar	6899.	6592.85	{6503., 6684.}	4.4376
19	R0	{1.70787, 9.85478, 0.1}	{1.84021, 13.0803, 2.}	{{1.84021, 13.0803, 2.}, {1.84021, 13.0803, 2.}}	{7.7489, 32.7309, 1900.}	inf	473.	599.389	{574.748, 620.317}	26.7207	0.102683	newly	313.	292.16	{271., 314.}	7.29712	0.0333652	Reff	0.632595	0.658356	{0.654467, 0.662268}	4.07226	ar	6867.	7238.05	{7140., 7332.}	5.40338
20	R0	{1.53809, 10.5848, 1.}	{1.41143, 6.58664, 2.62}	{{1.39162, 3.22945, 1.}, {1.41695, 7.40677, 3.}}	{8.23496, 37.7727, 178.}	inf	613.	258.325	{152.202, 309.213}	57.8588	0.394039	newly	373.	246.4	{206., 279.}	34.1877	0.187417	Reff	0.804882	0.7886	{0.782815, 0.793339}	2.1079	ar	6108.	5113.77	{4730., 5325.}	16.2775
21	R0	{1.59925, 6.74479, 0.1}	{1.54522, 5.29627, 3.99}	{{1.54465, 5.30296, 4.}, {1.54465, 5.30296, 4.}}	{3.37828, 21.4762, 3890.}	inf	243.	160.464	{138.666, 184.639}	33.9655	0.182955	newly	202.	167.74	{144., 200.}	17.7525	0.0876088	Reff	0.649775	0.640578	{0.63655, 0.643708}	1.44502	ar	6411.	6111.51	{6037., 6182.}	4.6715
22	R0	{1.38779, 4.40977, 0.1}	{1.44112, 5.71846, 0.}	{{1.44485, 6.00596, 0.}, {1.44485, 6.00596, 0.}}	{3.84292, 37.6488, 100.}	inf	176.	261.474	{230.028, 306.399}	55.8161	0.202704	newly	221.	272.77	{238., 314.}	25.6425	0.0993441	Reff	0.751211	0.767695	{0.76225, 0.772597}	2.19442	ar	5154.	5330.21	{5189., 5502.}	4.52018
23	R0	{1.53657, 7.50352, 1.}	{1.44485, 6.00596, 0.}	{{1.44485, 6.00596, 0.}, {1.44485, 6.00596, 0.}}	{5.96931, 19.9581, 100.}	inf	330.	202.698	{172.603, 229.64}	38.5762	0.214506	newly	276.	200.08	{165., 230.}	27.5072	0.143048	Reff	0.757222	0.730414	{0.726439, 0.734686}	3.54028	ar	5928.	5410.45	{5288., 5515.}	8.7306
24	R0	{1.71131, 6.24526, 0.1}	{1.69756, 6.15907, 2.}	{{1.69756, 6.15907, 2.}, {1.69756, 6.15907, 2.}}	{0.803188, 1.3801, 1900.}	inf	308.	296.96	{264.1, 322.551}	6.40976	0.0289728	newly	276.	268.68	{241., 293.}	6.68116	0.0301406	Reff	0.599814	0.596871	{0.591774, 0.601718}	0.673567	ar	6924.	6863.01	{6764., 6938.}	1.02065
25	R0	{1.4412, 3.07729, 0.1}	{1.4804, 3.12918, 2.}	{{1.4804, 3.12918, 2.}, {1.4804, 3.12918, 2.}}	{2.71978, 1.6862, 1900.}	inf	56.	58.5326	{27.5272, 88.3835}	32.704	0.158637	newly	93.	90.48	{42., 134.}	31.1183	0.161943	Reff	0.624328	0.640933	{0.63651, 0.645518}	2.65973	ar	5810.	5775.72	{5691., 5851.}	1.03339
26	R0	{1.4159, 4.93858, 0.1}	{1.41608, 4.91936, 1.98}	{{1.41571, 4.94504, 2.}, {1.41571, 4.94504, 2.}}	{0.0766231, 0.642771, 1884.}	inf	215.	172.452	{144.936, 198.657}	20.0865	0.101445	newly	264.	203.24	{169., 238.}	23.0606	0.118107	Reff	0.740657	0.74678	{0.742366, 0.749969}	0.841223	ar	5255.	5162.01	{5005., 5284.}	2.16537
27	R0	{1.57465, 7.05414, 0.1}	{1.54465, 5.30296, 4.}	{{1.54465, 5.30296, 4.}, {1.54465, 5.30296, 4.}}	{1.90515, 24.8249, 3900.}	inf	430.	290.303	{255.265, 320.741}	32.4876	0.172452	newly	370.	315.5	{278., 354.}	15.2162	0.0735629	Reff	0.69946	0.707712	{0.702556, 0.713015}	1.18712	ar	6359.	5984.04	{5848., 6137.}	5.89652
28	R0	{1.41422, 4.62402, 0.1}	{1.4509, 4.01074, 0.}	{{1.44762, 4.00571, 0.}, {1.47488, 4.06661, 0.}}	{2.59391, 13.263, 100.}	inf	181.	133.194	{96.5512, 162.201}	26.4124	0.142377	newly	219.	181.12	{134., 231.}	19.8995	0.103253	Reff	0.681654	0.706269	{0.700474, 0.713723}	3.61102	ar	5485.	5424.92	{5305., 5569.}	1.7783
29	R0	{1.41901, 3.63017, 0.1}	{1.53501, 4.02511, 0.}	{{1.53501, 4.02511, 0.}, {1.53501, 4.02511, 0.}}	{8.17462, 10.8793, 100.}	inf	109.	111.123	{83.5365, 137.494}	15.315	0.067516	newly	157.	142.96	{108., 178.}	16.4204	0.0793525	Reff	0.594707	0.642996	{0.63878, 0.647186}	8.11983	ar	5979.	5996.87	{5911., 6092.}	0.97625
30	R0	{1.60719, 4.72984, 0.1}	{1.63764, 4.36615, 2.8}	{{1.61691, 4.30394, 2.}, {1.64953, 4.41442, 4.}}	{1.89487, 8.23103, 2700.}	inf	159.	156.604	{124.754, 186.255}	12.5447	0.0549131	newly	176.	184.09	{141., 226.}	14.4034	0.060107	Reff	0.595464	0.607131	{0.597747, 0.615355}	1.98236	ar	6479.	6511.6	{6417., 6597.}	0.956938
31	R0	{1.38175, 6.26468, 0.1}	{1.43029, 4.08388, 0.78}	{{1.39162, 2.60601, 0.}, {1.44762, 4.94504, 2.}}	{3.52462, 34.811, 802.}	inf	255.	123.432	{73.6534, 159.108}	51.5953	0.344321	newly	236.	168.67	{121., 202.}	29.2331	0.166242	Reff	0.70939	0.753117	{0.739697, 0.765005}	6.16398	ar	5397.	5057.56	{4899., 5211.}	6.28942
32	R0	{1.4947, 8.8847, 1.}	{1.39507, 7.47062, 3.}	{{1.39507, 7.47062, 3.}, {1.39507, 7.47062, 3.}}	{6.66543, 15.9159, 200.}	inf	294.	173.204	{151.233, 193.321}	41.0872	0.231656	newly	208.	143.18	{122., 163.}	31.1635	0.164851	Reff	0.787707	0.752055	{0.748913, 0.755064}	4.52606	ar	5407.	5054.38	{4926., 5199.}	6.52155
33	R0	{1.55891, 8.28978, 1.}	{1.51295, 8.4085, 1.}	{{1.51295, 8.4085, 1.}, {1.51295, 8.4085, 1.}}	{2.94825, 1.43209, 0.}	inf	374.	277.138	{254.308, 300.281}	25.8989	0.131131	newly	292.	203.8	{182., 228.}	30.2055	0.157846	Reff	0.720061	0.713486	{0.709792, 0.716855}	0.91389	ar	6239.	5851.87	{5735., 5965.}	6.205
34	R0	{1.60764, 10.4729, 0.1}	{1.99038, 17.2142, 3.}	{{1.99038, 17.2142, 3.}, {1.99038, 17.2142, 3.}}	{23.8079, 64.3685, 2900.}	inf	407.	918.015	{889.961, 944.647}	125.556	0.353151	newly	229.	353.37	{332., 376.}	54.31	0.187906	Reff	0.67971	0.739823	{0.734465, 0.745064}	8.84389	ar	6495.	7628.54	{7526., 7733.}	17.4525
35	R0	{1.48527, 5.74485, 1.}	{1.45537, 4.11887, 0.21}	{{1.44762, 4.00571, 0.}, {1.47488, 4.06661, 0.}}	{2.01328, 28.3032, 107.}	inf	235.	141.346	{104.521, 182.871}	40.1424	0.234382	newly	239.	185.56	{138., 232.}	23.8912	0.125684	Reff	0.703869	0.703309	{0.697081, 0.711199}	0.652739	ar	5811.	5471.88	{5344., 5607.}	5.85407
36	R0	{1.61457, 9.93364, 1.}	{1.56097, 8.77207, 2.}	{{1.56097, 8.77207, 2.}, {1.56097, 8.77207, 2.}}	{3.31968, 11.6933, 100.}	inf	545.	482.747	{451.812, 509.491}	11.4225	0.0531064	newly	332.	342.91	{317., 365.}	5.59337	0.0236629	Reff	0.74577	0.73073	{0.726547, 0.735449}	2.01667	ar	6338.	6250.57	{6115., 6377.}	1.66409
37	R0	{1.47831, 7.08139, 0.1}	{1.44485, 6.00596, 0.}	{{1.44485, 6.00596, 0.}, {1.44485, 6.00596, 0.}}	{2.26358, 15.1867, 100.}	inf	340.	250.936	{212.45, 280.897}	26.1954	0.134299	newly	278.	251.61	{212., 287.}	11.3561	0.0538943	Reff	0.759556	0.755231	{0.750892, 0.760737}	0.643056	ar	5530.	5388.5	{5208., 5523.}	2.80579
38	R0	{1.73445, 9.8858, 0.1}	{1.58886, 7.29751, 1.}	{{1.58886, 7.29751, 1.}, {1.58886, 7.29751, 1.}}	{8.39376, 26.1819, 900.}	inf	569.	330.632	{302.152, 358.944}	41.8925	0.236765	newly	367.	268.76	{239., 293.}	26.7684	0.136775	Reff	0.688056	0.668176	{0.663574, 0.672386}	2.88934	ar	6842.	6328.64	{6216., 6464.}	7.50307
39	R0	{1.66621, 11.4824, 0.1}	{1.51295, 8.4085, 1.}	{{1.51295, 8.4085, 1.}, {1.51295, 8.4085, 1.}}	{9.19815, 26.7706, 900.}	inf	533.	306.623	{279.206, 334.98}	42.4722	0.241188	newly	292.	225.05	{201., 250.}	22.9281	0.114714	Reff	0.756459	0.721129	{0.716646, 0.725196}	4.67052	ar	6473.	5870.08	{5734., 5981.}	9.31438
40	R0	{1.60694, 9.95741, 1.}	{1.56097, 8.77207, 2.}	{{1.56097, 8.77207, 2.}, {1.56097, 8.77207, 2.}}	{2.86063, 11.9041, 100.}	inf	510.	423.792	{398.363, 448.515}	16.9035	0.0808732	newly	314.	295.93	{271., 317.}	6.57006	0.0299321	Reff	0.690181	0.683894	{0.679935, 0.687804}	0.920933	ar	6625.	6316.33	{6219., 6418.}	4.65917
41	R0	{1.55128, 4.71911, 0.1}	{1.54405, 4.40031, 1.59}	{{1.53501, 4.02511, 0.}, {1.55618, 4.83753, 4.}}	{0.699472, 8.61235, 1592.}	inf	233.	176.072	{132.72, 223.399}	24.8966	0.133549	newly	259.	213.17	{172., 257.}	19.0309	0.0968825	Reff	0.655881	0.661591	{0.655479, 0.666246}	0.937129	ar	6141.	6017.36	{5887., 6152.}	2.20909
42	R0	{1.60063, 6.81118, 0.1}	{1.70783, 8.50869, 2.84}	{{1.70777, 8.49961, 3.}, {1.70777, 8.49961, 3.}}	{6.69731, 24.9224, 2740.}	inf	295.	476.511	{451.222, 506.015}	61.529	0.207816	newly	240.	334.41	{307., 364.}	39.3375	0.143296	Reff	0.619924	0.630443	{0.625384, 0.634503}	1.69675	ar	6528.	6942.85	{6850., 7025.}	6.35493
43	R0	{1.56986, 5.44652, 0.1}	{1.58156, 5.07636, 3.85}	{{1.55618, 4.83753, 3.}, {1.59034, 5.09935, 4.}}	{1.26388, 6.79619, 3750.}	inf	202.	164.34	{134.903, 192.005}	18.8299	0.0947044	newly	196.	176.43	{145., 210.}	13.5459	0.0652604	Reff	0.625118	0.635716	{0.628151, 0.641717}	1.76366	ar	6312.	6241.3	{6131., 6312.}	1.26648
44	R0	{1.47523, 8.07785, 0.1}	{1.41221, 5.80414, 0.76}	{{1.40191, 5.7404, 0.}, {1.44485, 6.00596, 1.}}	{4.27164, 28.1475, 708.}	inf	323.	196.994	{158.466, 243.321}	39.0111	0.220016	newly	237.	202.98	{166., 249.}	16.7679	0.082261	Reff	0.760776	0.746033	{0.738558, 0.757366}	1.9531	ar	5692.	5190.84	{5018., 5368.}	8.80604
45	R0	{1.6417, 7.69429, 0.1}	{1.67512, 7.31377, 1.12}	{{1.67405, 7.28757, 1.}, {1.68297, 7.5059, 2.}}	{2.03568, 4.94544, 1020.}	inf	363.	399.32	{368.64, 430.731}	10.1833	0.0414267	newly	273.	316.15	{291., 342.}	15.8059	0.0628817	Reff	0.605951	0.612201	{0.6066, 0.617109}	1.05839	ar	6735.	6868.48	{6774., 6963.}	1.98456
46	R0	{1.38865, 5.83473, 0.1}	{1.40188, 5.61692, 0.99}	{{1.40191, 5.7404, 1.}, {1.40191, 5.7404, 1.}}	{0.974113, 3.733, 896.}	inf	305.	215.694	{178.67, 255.267}	29.2808	0.158473	newly	319.	233.6	{191., 269.}	26.79	0.139109	Reff	0.760702	0.780464	{0.774407, 0.785191}	2.59782	ar	5233.	5041.1	{4865., 5213.}	3.89031
47	R0	{1.45289, 6.87903, 0.1}	{1.41838, 5.1638, 1.47}	{{1.40191, 4.94504, 0.}, {1.44485, 6.00596, 2.}}	{2.37535, 24.9342, 1408.}	inf	301.	212.608	{169.561, 259.539}	29.4482	0.15651	newly	259.	244.89	{202., 287.}	10.7297	0.0496067	Reff	0.779475	0.773485	{0.763619, 0.781151}	1.0573	ar	5379.	5136.89	{4971., 5336.}	4.59249
48	R0	{1.69416, 9.4831, 0.1}	{1.58886, 7.29751, 1.}	{{1.58886, 7.29751, 1.}, {1.58886, 7.29751, 1.}}	{6.21521, 23.0472, 900.}	inf	573.	357.244	{326.657, 384.702}	37.6538	0.20623	newly	377.	292.67	{266., 316.}	22.3687	0.111139	Reff	0.683255	0.67507	{0.670437, 0.679878}	1.20515	ar	6925.	6340.97	{6233., 6451.}	8.43365
49	R0	{1.47843, 7.16286, 0.1}	{1.44615, 5.46483, 0.}	{{1.44485, 4.00571, 0.}, {1.44762, 6.00596, 0.}}	{2.18318, 23.706, 100.}	inf	252.	207.983	{138.517, 260.354}	18.7193	0.0986297	newly	214.	223.84	{184., 264.}	12.6075	0.0531544	Reff	0.752669	0.74261	{0.734296, 0.753412}	1.47864	ar	5449.	5361.25	{5153., 5516.}	2.31327
50	R0	{1.68843, 5.72427, 0.1}	{1.6964, 6.13541, 2.09}	{{1.69756, 6.15907, 2.}, {1.69756, 6.15907, 2.}}	{0.577713, 7.5533, 1990.}	inf	170.	227.354	{199.1, 253.004}	34.923	0.129244	newly	156.	204.46	{180., 228.}	31.8205	0.118658	Reff	0.581326	0.57433	{0.570331, 0.578804}	1.20361	ar	6800.	6893.17	{6829., 6970.}	1.45985
